# A multi-genotype therapeutic human papillomavirus vaccine elicits potent T cell responses to conserved regions of early proteins

**DOI:** 10.1038/s41598-019-55014-z

**Published:** 2019-12-10

**Authors:** Gemma Hancock, Joshua Blight, Cesar Lopez-Camacho, Jakub Kopycinski, Mamatha Pocock, Wendy Byrne, Michael J. Price, Phillip Kemlo, Ranoromanana Ionitiana Evans, Angela Bloss, Kathryn Saunders, Richard Kirton, Monique Andersson, Karin Hellner, Arturo Reyes-Sandoval, Lucy Dorrell

**Affiliations:** 1 Nuffield Department of Medicine, University of Oxford and Oxford NIHR Biomedical Research Centre, NDM Research Building, Old Road Campus, Oxford, UK; 2 The Jenner Institute, Nuffield Department of Medicine, University of Oxford, The Henry Wellcome Building for Molecular Physiology, Roosevelt Drive, Oxford, UK; 3 Direct Delivery Team, NIHR Clinical Research Network Thames Valley and South Midlands, Nuffield Orthopaedic Centre, Block 8, Oxford, OX3 7LD UK; 4 Microbiology Department, Oxford University Hospital NHS Foundation Trust, Oxford, UK; 5 Nuffield Department of Obstetrics and Gynaecology, University of Oxford, Women’s Centre, John Radcliffe Hospital, Oxford, UK

**Keywords:** Cancer, Viral infection, Cervical cancer, Immunology

## Abstract

Despite an efficacious prophylactic human papillomavirus (HPV) vaccine there is still a considerable global burden of HPV-related disease. Therapeutic vaccines that could prevent cancers in at-risk women are urgently needed. Most candidate therapeutic vaccines have focused on two high-risk (hr) HPV genotypes, 16 and 18, and two viral targets, E6 and E7, which may limit global coverage and efficacy. We designed the synthetic gene ‘5GHPV3′ by selecting conserved regions from each of the six early proteins and generating consensus sequences to represent five hrHPV genotypes. 5GHPV3 was delivered by plasmid DNA, chimpanzee adenovirus (ChAdOx1) and modified vaccinia Ankara (MVA) vectors in prime-boost regimens to mice. ChAdOx1-5GHPV3 / MVA-5GHPV3 induced higher magnitude and more durable HPV-specific T cell responses than other regimens. Vaccine-induced T cells were polyfunctional and persisted at high frequencies for at least six weeks. Importantly, HPV-specific effector CD8 + T cells were detected in the cervix following systemic administration of ChAdOx1-5GHPV3 / MVA-5GHPV3 and increased in frequency over time, indicating continued trafficking of T cells to the cervix. Finally, T cells specific for 5GHPV3 encoded antigens were detected by IFN-γ Elispot in women with current or past hrHPV infections, confirming the presence of epitopes relevant to natural immune control.

## Introduction

Cervical cancer accounted for over 300,000 deaths in women worldwide in 2018^1^. It is the consequence of persistent infection by high risk human papillomaviruses (hrHPV)^2^. The majority of HPV infections are transient and subclinical due to rapid immune clearance. Precancerous lesions (cervical intraepithelial neoplasia, CIN) develop in approximately 25% women with a 6-month persistent cervical infection and a small fraction of these progress to invasion^3–5^.

Prophylactic bivalent, quadrivalent and 9-valent hrHPV vaccines prevent persistent cervical infections through induction of type-specific neutralizing antibodies to the major capsid protein L1^6,7^. However, they have no therapeutic efficacy against pre-existing HPV infections or pre-malignant lesions and provide limited cross-protection^8–11^. By October 2016, 86 countries had included hrHPV vaccines as part of their national vaccination schedule, however, only 12 of these countries were LIC/LMIC^12^. In both HIC and LIC/LMIC that offer the hrHPV vaccine as part of their national vaccination schedule, coverage achieved has been highly variable. Some HIC such as France, USA, Japan and Denmark have yet to achieve even 50% coverage^13^. Collectively this leaves millions of women at risk of hr-HPV infection and associated disease.

In hrHPV-exposed women, cervical cancer can be prevented by surgical excision or ablation of precursor lesions. However, this is associated with a ~10% recurrence rate and two-fold increased risk of adverse pregnancy outcomes^14–16^. As T cell responses play a crucial role in spontaneous clearance of HPV infection and in regression of CIN^17–21^, therapeutic vaccination has been proposed as a strategy to prevent the progression of low-grade disease, induce regression of existing lesions and prevent recurrence following treatment. A recent randomised controlled phase 2b clinical trial provided proof of concept for this therapeutic approach, using a DNA vaccine encoding the oncoproteins E6 and E7 from HPV16 and HPV18 in women with high-grade lesions (CIN2/3) caused by these genotypes. In the per-protocol analysis, the primary endpoint of histopathological regression occurred in 49.5% of vaccinees compared with 30.6% of placebo recipients^22^. However, the translational potential of therapeutic vaccination has yet to be fully realised, as recombinant DNA is poorly immunogenic in humans, necessitating the use of electroporation or adjuvants. Greater clinical efficacy might be achieved with more potent delivery systems. Replication-deficient adenoviruses out-perform other vectors in the induction of transgene-specific T cells in humans, especially when delivered in heterologous prime-boost immunisation schedules with recombinant modified vaccinia Ankara (MVA) vaccines^23^.

The mixed results observed with other therapeutic vaccines indicates that the choice of viral targets and genotype coverage require further consideration. While E6 and E7 are highly expressed in high-grade lesions and cancers, targeting other early proteins such as E2 may be beneficial. E2 initiates and maintains viral replication and T cell responses to E2 are associated with regression of CIN and viral clearance^19,20,24,25^. E5 cooperates with E6 and E7 oncogenes to promote hyper-proliferation of infected cells and is therefore crucial for malignant progression^26,27^. HPV16 and HPV18 cause at least 70% of cervical cancers and thus have been the focus of most interventions to date^28,29^. The remaining 30% are caused by 12 other hr types, which have received less attention.

To address these issues, we developed a multi-genotype immunogen that comprises segments from each of the early proteins and is delivered by replication-deficient chimpanzee adenovirus and MVA vectors. This immunogen is predicted to provide coverage of 85% of circulating global hrHPV genotypes that cause cervical cancer and builds upon existing concepts focusing predominantly on E6/E7 from HPV16/18.

## Materials and Methods

### Vaccine design and construction

The 5GHPV3 immunogen was designed using conservation software developed in-house, written using the Java SE Development Kit 8 (Oracle). All available full-length sequences for HPV proteins E1, E2, E4, E5, E6 and E7 from genotypes 16, 18, 31, 52 and 58 of high quality were extracted from the NCBI Protein database (accessed 2014) and used as input for our approach (Supplementary Table S1). Prior to conservation analysis of each early protein, all genotypes were aligned using Clustal Omega (EMBL-EBI) multiple alignment tool with default settings. Sequences within each genotype were then weighted to account for sampling bias and thus ensure that our vaccine candidate was representative of the true environmental population. Conservation was subsequently assessed using our bespoke software, based on a sliding window approach. First, conservation within genotypes (intra-genotype) for each early protein was assessed using a 15 amino acid sliding window which assigns a conservation value (between 0–1) based on combining the amino acid prevalence within the window and weighting value of each sequence. Windows with a conservation value within the first quartile were then used to select regions for further analysis and to create a normalised consensus for each genotype (referred to as intra consensus). These intra consensus sequences were utilised to identify shared regions across genotypes (inter-genotype). In this case, inter-genotype conservation refers to windows at the same position across genotypes that were within the first quartile. Neighbour-joining trees were used to identified outgroups using the Jukes-Cantor model with 1000 retrials.

The gene encoding the 5GHPV3 immunogen was synthetically produced using humanised codons (GeneArt). The human tissue plasminogen activator leader sequence was added at the 5′ end to enhance expression^30,31^ and a Kozak sequence was included to improve the initiation of translation. The plasmid encoding 5GHPV3, as well as a pENTR4-Mono backbone plasmid were digested with KpnI/NotI. The pENTR4-Mono backbone plasmid was dephosphorylated and ligated with the 5GHPV3 fragment. pENTR4-Mono-5GHPV3 (DNA-5GHPV3) was expanded in Escherichia coli, purified by endotoxin-free giga prep (Qiagen) and verified by restriction analysis and 5′ and 3′ flanking sequencing. ChAdOx1-5GHPV3 was produced by *In vitro* recombination (LR Clonase II system; ThermoFisher Scientific) between pENTR4-Mono-5GHPV3 and the replication-defective chimpanzee adenovirus Ox1 (ChAdOx1) shuttle plasmid^32^. The resulting plasmid was linearised by PmeI restriction enzyme digestion. To generate the Modified Vaccinia Ankara (MVA) vaccines, the 5GHPV3 plasmid, as well as the MVA backbone plasmid were digested with KpnI/XhoI. The MVA backbone plasmid was dephosphorylated and ligated with 5GHPV3. The resulting plasmid was linearised by AatII restriction enzyme digestion. ChAdOx1-5GHPV3 was produced in T-Rex^TM^-293 cells (ThermoFisher Scientific) and MVA-5GHPV3 in chick embryo fibroblasts at the Viral Vector Core Facility (Jenner Institute, University of Oxford, Oxford, UK).

### Experimental animals and immunisations

Six-week-old female C57BL/6 and CD1 mice were purchased from Harlan, UK and Charles River, UK respectively. Mouse care and experimental procedures were carried out in accordance with the UK Animals (Scientific Procedures) Act under Project Licences P9804B4F1 (30/2889) and 30/2947 and were approved by the University of Oxford Animal Care and Ethical Review Committee. Mice were immunised with DNA-5GHPV3 (100 µg), ChAdOx1-5GHPV3 (1 × 10^8^ infectious units, IU) or MVA-5GHPV3 (1 × 10^6^ plaque forming units, pfu) either alone (‘D’, ‘C’ or ‘M’) or in prime-boost schedules (‘DD’, ‘CC’, ‘MM’, ‘DM’, ‘CM’). Vaccinations were performed via the intramuscular (i.m) route, in a final volume of 50 μl and injected into the tibialis anterior muscle of each animal (25 µl per anterior muscle). All viruses and DNA were resuspended in endotoxin-free PBS for immunization.

### Human study participants

Healthy non-pregnant women aged 16–55 years with current or prior hrHPV infection were recruited to an observational study of natural immune responses to hrHPV, which was approved by the South West - Central Bristol Research Ethics Committee (Ref. 16/SW/0331). All methods were performed in accordance with the relevant guidelines and regulations. Informed consent was obtained from all participants and / or their legal guardian. Peripheral blood and vaginal samples were obtained with written informed consent for the purposes of analysing T cell responses to hrHPV peptides in blood and the presence of hrHPV DNA in the vagina. Women aged 16–24 years were enrolled directly from the community if they were currently or previously sexually active (Cohort 1). Women aged 25–55 years who had been referred to Oxford University Hospitals NHS Trust Colposcopy for investigation of abnormal cervical cytology were enrolled if they had either HPV-related change, CIN1, CIN2 or CIN3 on colposcopic examination (Cohort 2). Women were provided with vaginal swabs for self-sampling. Women reporting sexual intercourse in the preceding 48 hours were asked not to self-sample until 48 hours had elapsed, to minimise detection of HPV from partners that may not represent a true infection. Women requiring excisional treatment for CIN were asked to self-sample either before treatment or 4 weeks later to minimise the risk of bleeding.

### HPV DNA assay

Vaginal samples were tested on the Cobas 4800 platform (Roche, Pleasanton, California) a DNA-based, FDA-approved assay that detects viral DNA of 12 high risk genotypes with concurrent genotyping of HPV-16 and HPV-18. All samples were tested in a UKAS accredited laboratory (Department of Microbiology, Oxford University Hospitals NHS Foundation Trust, Oxford)

### Peptides

For mouse immunisation studies, 20-mer peptides overlapping by 10 amino acids (aa) and representing the entire 5GHPV3 immunogen were synthesised by Pepscan (The Netherlands). Peptides were pooled according to protein source or by segment and used at a final concentration of 10 µg/ml. For analysis of human immune responses to the sequences in 5GHPV3, 15-mer peptides overlapping by 11 aa were synthesised to >80% purity by Genscript. In addition, peptides representing all early proteins from HPV16 and HPV52 were synthesised (15-mers overlapping by 11 aa, >80% purity, Genscript) and combined in pools of 23–143 peptides (E1/E2, E4/E5 and E6/E7 for each genotype).

### Mouse peripheral blood mononuclear cell (PBMC) isolation

A tail vein bleed was used to collect 200 µL blood in 10 mM EDTA/PBS. PBMC were isolated by lysis of red blood cells using ammonium-chloride-potassium (ACK) lysis buffer and resuspended in 100 µL R10 complete medium (RPMI supplemented with 10% foetal calf serum, 5 ml penicillin/streptomycin and 5ml L-glutamine) prior to use.

### Mouse splenocyte isolation

Spleens were harvested and single cell suspensions were obtained by passage through a sterile 70 µm cell strainer. Splenocytes were incubated in ACK lysis buffer to lyse red blood cells, washed and resuspended in R10 complete medium. After an overnight rest at 37 °C, 5% CO_2,_ splenocytes were counted manually to assess cell viability by Trypan blue dye exclusion.

### Mouse female reproductive tract processing

Excess adipose and unwanted tissue were removed from the isolated female reproductive tracts. The ovaries and fallopian tubes were excised and discarded leaving the cervix and vaginal regions. The cervicovaginal region was washed and digested by the addition of collagenase type VIII (Sigma) at a final concentration of 1 mg/ml. Samples were placed on a MACSmix rotator at 37 °C for 30 minutes. The tissue was further disrupted using a 1 ml pipette tip prior to collection through a 70 µm cell strainer. To block further digestion, 10 ml of R15 medium (RPMI supplemented with 15% foetal calf serum, 5 ml penicillin/streptomycin and 5 ml L-glutamine) was added to each filtered cell suspension and samples were placed on ice. The remaining tissue was subjected to two further rounds of digestion following the same protocol. The cervicovaginal cells were resuspended in R10 complete medium and rested overnight.

### Human PBMC isolation

PBMC separation was performed by density centrifugation from whole blood within 6 hours of sampling. Cells were resuspended in R10 complete medium containing batch-tested foetal calf serum previously screened for low reactivity. Samples were stored in vapour phase liquid nitrogen until use.

### Mouse and human IFN-γ Elispot assays

Elispot plates (MSIPS 4510, Millipore) were coated with 5 µg/ml of anti-mouse IFN-γ (clone AN18, MabTech) capture antibody in the dark at 4 °C overnight. Plates were blocked with R10 complete medium at 37 °C for a minimum of 2 hours. Mouse PBMCs or splenocytes were incubated with 5GHPV3 peptide pools or individual peptides at a final concentration of 10 µg/ml. PMA/ionomycin at 5 μg/ml, and R10 (0.1% DMSO) were used as positive and negative control, respectively. Plates were incubated for 16–18 hours at 37 °C, after which a biotinylated anti-mouse IFN-γ detection antibody was added for 2 hours at room temperature. Streptavidin alkaline phosphatase polymer (Mabtech) was added for 1 hour at room temperature and the plates developed with 50 µL of 1-Step NBT/BCIP Substrate Solution (ThermoFisher Scientific) for 5 to 10 minutes until visible spots were observed on the membrane. Spot-forming units were counted using an automated reader (AID). The same procedure was used for human IFN-γ Elispot assays, apart from the use of anti-human IFN-γ coating antibody (clone 1-D1K, MabTech) and a biotinylated anti-human IFN-γ detection antibody (clone 7-B6-1, MabTech). The final peptide concentration for all the pools was 2 µg/ml. The assay cut-off for defining a positive response was 25 SFU/million (derived from mean + 3 SD of mock-stimulated well values).

### Intracellular cytokine staining

Following PBMC/splenocyte isolation from vaccinated mice, cells were rested overnight in R10 medium at 37 °C in a humidified incubator, then stimulated with 5GHPV3 peptide pools E1, E2, E4/5, E6 and E7 (10 µg/ml), negative control (0.1% DMSO) and positive controls (SEB, 10 µg/ml). Only the two immunodominant pools, E6 and E7 were used when cell number was limiting. Plates were incubated at 37 °C, 5% CO_2_ for 90 minutes with CD107a, after which 1 µL of both GolgiStop and GolgiPlug (BD Bioscience) were added to all wells for 5 hours. Plates were then stored in the dark at 4 °C overnight. Wells were resuspended in 100 µL mouse CD16/CD32 F_c_ block (BD Bioscience) and incubated in the dark, at 4 °C for 10 minutes. Following viability and surface staining, cells were then fixed using BD cytofix/cytoperm (BD Biosciences) and intracellularly stained (Supplementary Table S2). At least 10,000 viable singlet CD3+ CD4+ or CD8+ lymphocyte events were acquired using a CyAn flow cytometer (Supplementary Fig. S1). Data were analysed using FlowJo v9.9.3 (FlowJo, US) and GraphPad Prism v7.0.

### Statistical analysis

Graphing and statistical analysis were performed using GraphPad Prism version 7.00. One-way analysis of variance (ANOVA), followed by Tukey’s multiple comparisons test or two-way ANOVA followed by Sidak’s multiple comparison were performed. Results are indicated as follows: *p ≤ 0.05, **p ≤ 0.01, ***p ≤ 0.001, ****p ≤ 0.0001

## Results

### Design of a multi-protein multi-genotype immunogen using in silico analysis of highly conserved sequences

**Figure 1:**
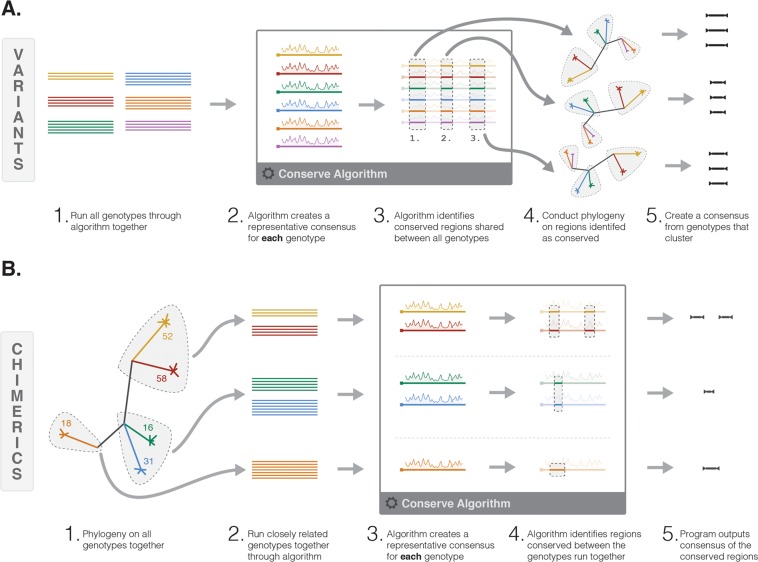
Schematic illustrating the variant and chimeric approach to HPV conservation-based vaccine design. (**A**) Variant immunogen design. (**B**) Chimeric immunogenic design. Colour lines represent HPV protein sequences, colours indicate different genotypes.

Our bespoke conservation software was utilised in two different ways, dependent on the level of shared conservation for each protein between genotypes to produce either (i) variants or (ii) chimerics (Fig. 1). Our default approach was to create variants (Fig. 1a). For this approach, all genotypes were run through the conservation algorithm together using a 15 amino acid sliding window. This identified windows which were conserved within each genotype (intra-genotype conservation), using a cut-off defined by the program. A normalised consensus sequence was then created for each window (or set of windows) that were conserved, referred to as intra consensus. Subsequently regions (one or more adjacent windows) which were conserved at the same window position across genotypes were identified (inter-genotype conservation). For each inter-genotype conserved region a neighbour joining tree was created using the intra consensus sequences and a consensus created from genotypes that clustered (final product).

Due to the unique phylogenetic clustering of HPV proteins, in some instances the distance between genotypes was so significant that genotypes did not align suitably. Therefore, the approach was modified and prior to analysis a neighbour-joining tree was created first. Genotypes which clustered were then aligned and run through the algorithm together and as before, intra-genotype and inter-genotype conservation was assessed and a final consensus created. The resulting fragments were referred to as chimerics (Fig. 1b).

Analysis of the entire HPV proteome identified 23 regions with conservation shared between multiple genotypes and enabled creation of 59 conserved fragments comprising 17 variants and 42 chimerics. These fragments totalled 1283 amino acids with an average fragment length of 21 amino acids (range 8–54aa). There were 11 fragments from E1, 24 from E2, 9 from E4, 3 from E5, 8 from E6 and 4 from E7. When totalling the number of amino acids for each protein, E1 constituted 16% of the immunogen, E2 36%, E4 13%, E5 6%, E6 18% and E7 10%. Fragments were directly combined in numerical order to create the 5GHPV3 immunogen (Patent number WO2019034887).

### Vaccination with heterologous ChAdOx1-5GHPV3 prime MVA-5GHPV3 boost induces strong T cell responses in inbred mice

To assess the immunogenicity of 5GHPV3 in inbred mice using various prime-boost regimens, groups of six C57BL/6 female mice were primed with either DNA-5GHPV3, MVA-5GHPV3 or ChAdOx1-5GHPV3 and then boosted with a heterologous or homologous vector vaccine two weeks later.

**Figure 2:**
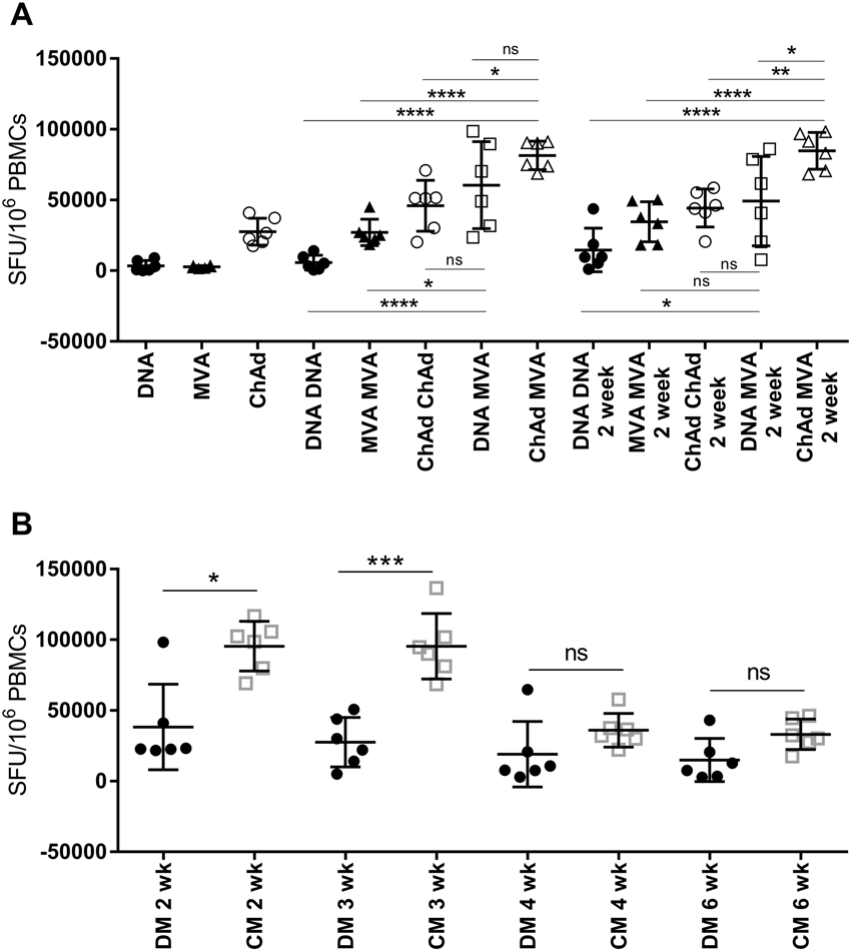
Induction of T cell responses to 5GHPV3 administered in different vaccination regimens, as measured by IFN-γ Elispot. C57BL/6 mice (6/group) were primed intramuscularly with DNA-5GHPV3 (100 µg), MVA-5GHPV3 (1 × 10^6^ pfu) or ChAdOx1-5GHPV3 (1 × 10^8^ IU) and then boosted intramuscularly with a heterologous or homologous vaccine two weeks later. Tail vein bleeds were performed two weeks post-prime and one and two weeks post-boost. PBMCs were stimulated with peptides spanning the entire immunogen sequence, pooled according to protein source. Data presented is (**A**) summed responses to all the pools. Further tail vein bleeds were done at three, four and six weeks post-boost immunisation (**B**). Horizontal lines show mean with standard deviation. One-way ANOVA with Tukey’s multiple comparison testing. *p ≤ 0.05, **p ≤ 0.01, ***p ≤ 0.001, ****p ≤ 0.0001.

We detected 5GHPV3-specific T cell responses after a single immunisation with either DNA, MVA or ChAdOx1 vectors. The highest number of HPV-specific T cells was elicited by ChAdOx1 vectored 5GHPV3; mean (SD) 27574 (9506) SFU/million PBMCs. DNA and MVA-vectored 5GHPV3 induced mean (SD) responses of 3451 (3818) and 2722 (944) SFU/million PBMCs respectively. Mice immunised with a MVA-unrelated, ChAdOx1-unrelated or empty DNA vaccine all gave transgene-specific responses below 100 SFU/million PBMCs. The magnitude of T cell responses induced by all three prime vaccinations was increased following homologous and heterologous boost vaccinations. However, the CM regimen elicited the strongest response by two weeks post-boost (mean (SD) total responses of 84736 (13022) SFU/million PBMCs) and this was significantly higher than vaccination with any other heterologous or homologous regimen (p < 0.0001; Fig. 2A). Responses were directed predominantly towards E6 ≥ E7 > E4/5 following all vaccination regimens (Supplementary Fig. S2). In CM- immunised mice, low magnitude responses to E1 and E2 were detected, although at higher frequency than in mice vaccinated with DD, MM, CC or DM (mean 2775 vs 295, 890, 659, 1539 SFU/million PBMCs respectively).

Peak HPV-specific T cell responses were observed two weeks post-boost for all homologous and heterologous prime-boost regimens apart from DM, for which this occurred at one week. Substantial numbers of HPV-specific T cells were still detectable six weeks post-boost in CM-immunised mice (mean (SD): 33117 (10764) SFU/million PBMCs, Fig. 2B) and the immunodominance hierarchy was unchanged at this point.

### T cells induced by 5GHPV3 vaccination are polyfunctional and produce cytokines associated with cytotoxicity

**Figure 3:**
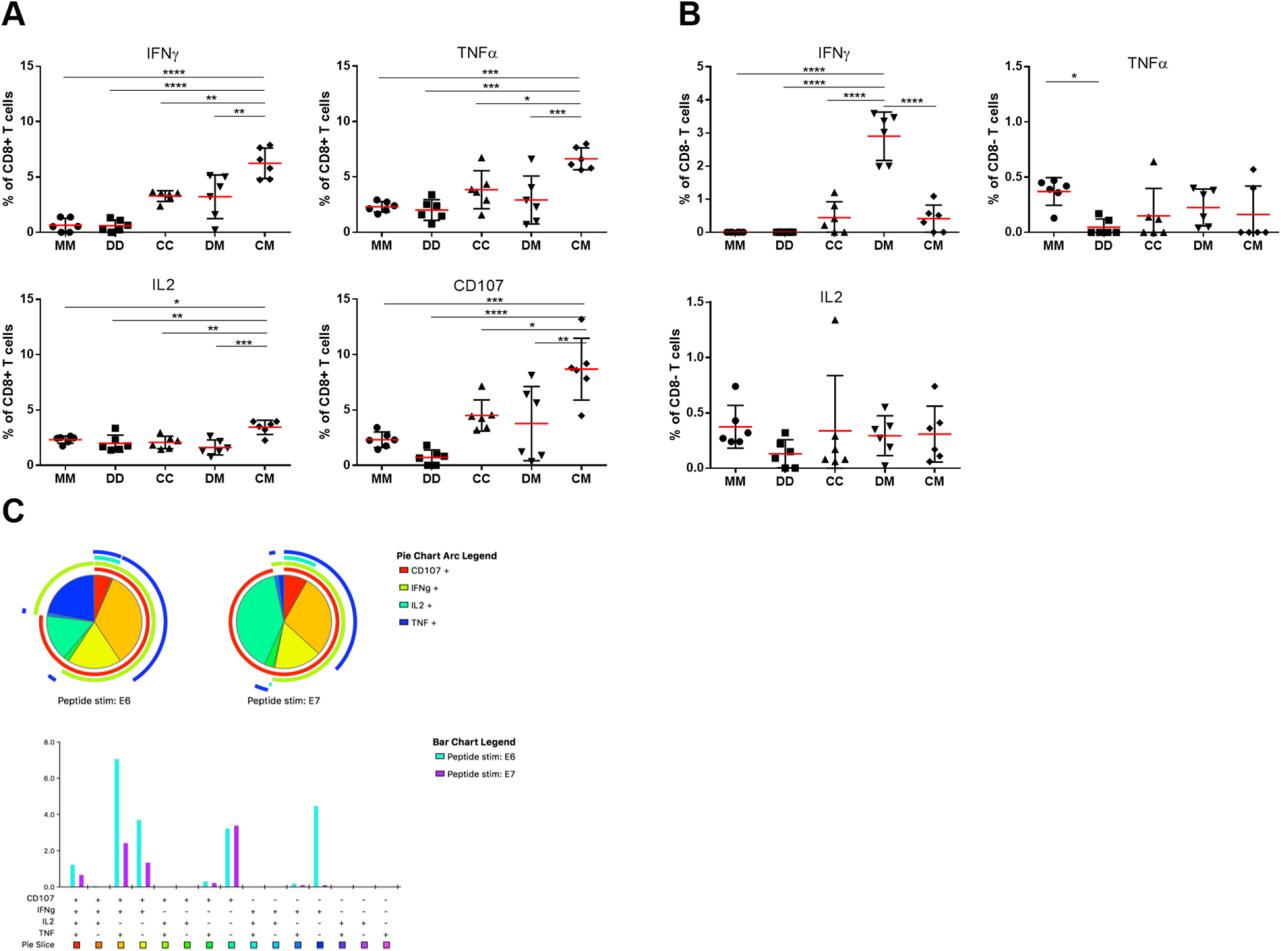
Functional characterisation of 5GHPV3-specific T cells. C57BL/6 mice (6/group) were vaccinated with MVA-5GHPV3 prime and boost (MM), DNA-5GHPV3 prime and boost (DD), ChAdOx1-5GHPV3 prime and boost (CC), DNA-5GHPV3 prime MVA-5GHPV3 boost (DM) or ChAdOx1-5GHPV3 prime MVA-5GHPV3 boost (CM). Splenocytes harvested two weeks post-boost were stimulated with peptides spanning the entire immunogen sequence, pooled according to protein source and analysed by intracellular staining (ICS) for IFN-ɣ, CD107, TNF-α and IL-2. Data shown is (**A**) E6-specific CD8+ T cell responses and (**B**) E6-specific CD8^−^ (CD4+) T cell responses. (**C**) ICS was performed on PBMC from C57BL/6 mice (6/group) vaccinated with the CM regimen at one week post-boost following stimulation with 5GHPV3 E6 and E7 peptide pools. Polyfunctionality of E6/E7-specific CD8+ T cells was determined using Pestle and Spice software. Horizontal lines show mean with standard deviation. One-way ANOVA, Tukey’s multiple comparison test. *p ≤ 0.05, **p ≤ 0.01, ***p ≤ 0.001, ****p ≤ 0.0001.

We performed intracellular cytokine staining (ICS) on splenocytes isolated two weeks post-boost in mice vaccinated with prime-boost regimens to assess the expression of IFN-γ, TNF-α, IL-2 and CD107a in response to stimulation with peptides spanning the entire transgene. Consistent with the Elispot data, the CM regimen elicited the highest frequency of E6-specific IFN-γ CD8+ T cells (mean (SD) 6.2% (1.4%) of total CD8+ T cells, Fig. 3A). TNF-α+, IL-2+ and CD107a + cells were also significantly enriched in HPV-specific CD8+ T cells in CM-immunised mice compared with the other regimens (P < 0.0001, 0.0003 and <0.0001, Fig. 3A). HPV-specific CD4+ T cells (CD3+ CD8- gated population) were also detected following CM vaccination but accounted for a smaller fraction of the response (mean (SD) 0.4% (0.4%) of total CD4+ T cells in spleens isolated two weeks post-boost, Fig. 3B). We used Boolean gating to calculate proportions of monofunctional and polyfunctional T cells. This showed that a high proportion of HPV-specific CD8+ T cells simultaneously expressed IFN-γ, TNF-α and CD107a, an indicator of cytotoxic potential (Fig. 3C). We also compared responses to E6 and E7 at one and six weeks post-boost: although IFN-γ+, TNF-α+ and CD107+ CD8+ T cell populations had contracted during this time they were still present at high frequency (Supplementary Fig. S3a). IL-2+ CD8+ T cells had increased by week six, consistent with the development of a memory response (for E6 from 1.3% to 3.1% and for E7 from 0.6% to 0.8%). As observed with HPV-specific CD8+ T cells in the periphery, the number of HPV-specific CD4^+^ IFN-γ+ T cells declined between one and six weeks post-boost from 2.6% to 0.7% but the percentage of IL-2-producing HPV-specific CD4+ T cells remained constant (0.7% vs 0.6%), indicating continued CD4^+ ^T cell help (Supplementary Fig. S3b).

### HPV-specific effector T cells traffic to the cervix following systemic immunisation

**Figure 4:**
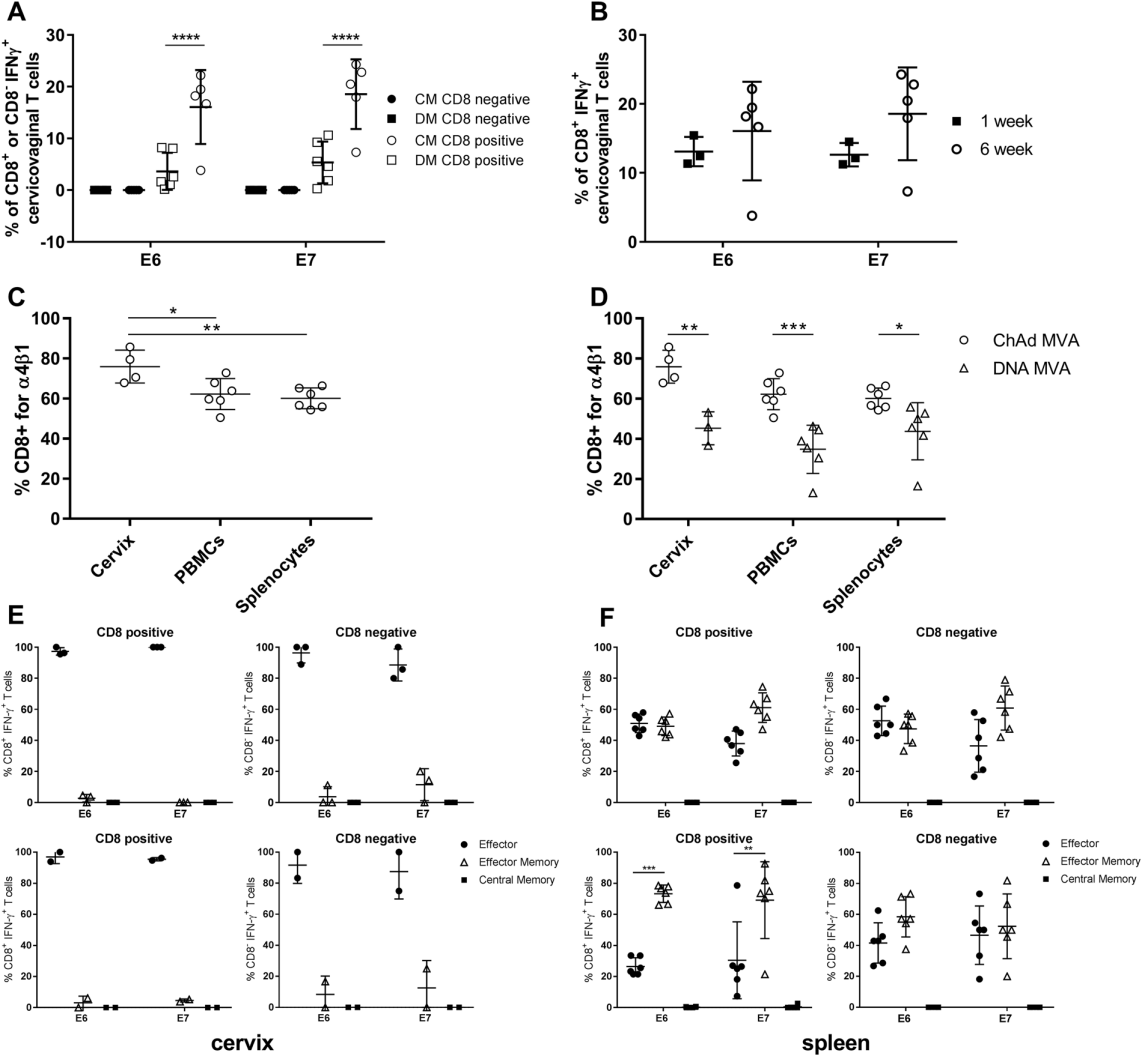
5GHPV3-specific CD8 + T cells with an effector phenotype are detected in the cervix following administration of heterologous prime-boost vaccine regimens. (**A**) C57BL/6 mice (6/group) were vaccinated with DM or CM regimens. Cervicovaginal lymphocytes were isolated six weeks post-boost, stimulated with 5GHPV3 E6 and E7 peptides and analysed by ICS for IFN-ɣ, CD107, TNF-α and IL-2. (**B**) For CM-vaccinated mice, cervicovaginal lymphocyte responses were compared at one and six weeks post-boost. Mice were pooled due to low cell numbers. (**C**) Comparison of CD49d/CD29 (α4β1) expression in 5GHPV3 E6-specific T cells among cervicovaginal lymphocytes, splenocytes and PBMCs at two weeks post-boost in CM-vaccinated C57BL/6 mice and (**D**) in CM vs. DM vaccinated C57BL/6 mice. Expression of differentiation/memory markers in 5GHPV3 E6 or E7-specific T cells was assessed in (**E**) cervicovaginal lymphocytes and (**F**) splenocytes from C57BL/6 mice (6/group) vaccinated with CM at one week (top panels) and three weeks (bottom panels) post-boost. Samples were pooled due to low cell number. Effector (CD62L^−^, CD127^−^), effector memory (CD62L^−^, CD127+) and central memory (CD62+, CD127+) populations are shown. Horizontal lines show mean with standard deviation. Two-way ANOVA, Sidak’s multiple comparison test. *p ≤ 0.05, **p ≤ 0.01, ***p ≤ 0.001, ****p ≤ 0.0001.

Since induction of potent and durable immune responses in the female genital tract is necessary for interception of hrHPV disease, this was assessed following vaccination of mice with the two heterologous vaccination regimens, DM and CM. Six weeks post-boost, cervicovaginal lymphocytes were isolated by digestion of the cervicovaginal tissue with collagenase. A mean (SD) of 46842 (13030) CD3+ lymphocytes were isolated from each cervicovaginal region. Sixteen per cent and 18% of cervicovaginal CD8+ T cells isolated from mice were specific for E6 and E7 peptide pools respectively in the CM group, compared with 3% and 5% in the DM group (Fig. 4A). The same trend, although not significant, was observed for CD107+ cervicovaginal CD8+ T cells. HPV-specific CD4+ T cells were also detected in the cervicovaginal region following both vaccination regimens although at a lower frequency (0.02% for CM and 0.003% for DM, Fig. 4A).

Further analysis of the kinetics of HPV-specific T cell responses in the cervicovaginal region showed that the frequency of HPV-specific CD8+ T cells increased from 12 to 18% between one and six weeks post-boost in the CM group, indicating continued trafficking of HPV-specific cells from the periphery to the cervicovaginal region (Fig. 4B). Consistent with this, a significantly higher percentage of vaccine-specific cervicovaginal CD8+ T cells expressed α4β1 integrin (CD49d/CD29), a mucosal homing marker, as compared with HPV-specific PBMCs and splenocytes (p = 0.0094, Fig. 4C). Additionally, following the CM regimen the percentage of HPV-specific cells expressing α4β1 was significantly higher in all compartments (cervicovaginal, blood and spleen) than with the DM regimen (p < 0.0001, Fig. 4D). CD4+ and CD8+ HPV-specific cervicovaginal lymphocytes were almost exclusively of an effector phenotype (CD62L^−^, CD127^−^), at both one and three weeks post-boost (Fig. 4E). By contrast, HPV-specific CD4+ and CD8+ cells from the spleen were of a mixed effector and effector memory phenotype (CD62L^−^, CD127+) and the latter increased over time (Fig. 4F). As was the case with HPV-specific splenocytes and PBMCs, HPV-specific cervicovaginal lymphocytes were polyfunctional, with the majority of CD8+ cells expressing IFN-ɣ, CD107, and TNFα.

### T cell responses in outbred mice elicited by heterologous prime-boost regimens target epitopes across the entire 5GHPV3 immunogen

**Figure 5:**
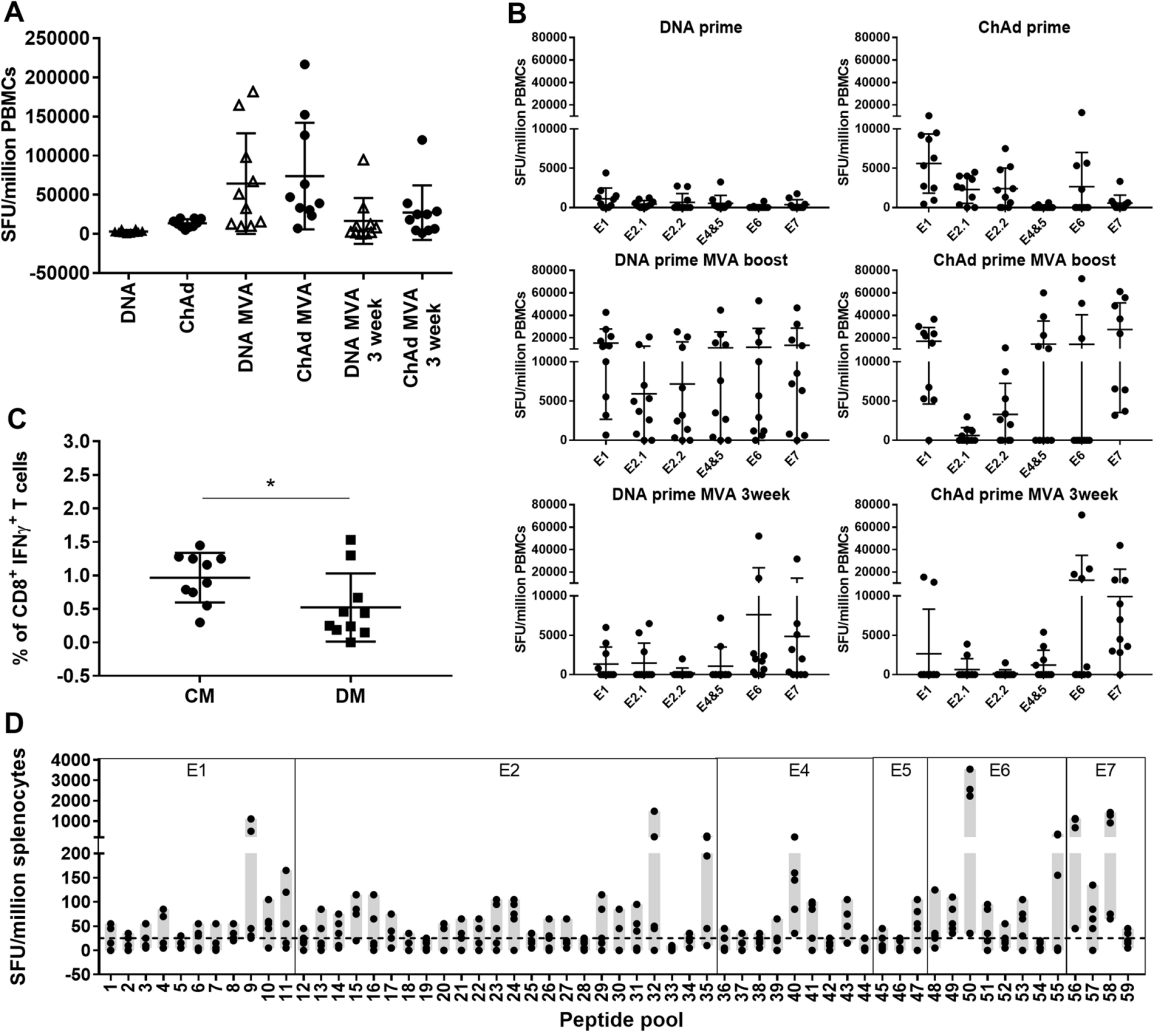
T cell responses induced by heterologous prime-boost vaccinations in outbred mice target nearly the entire 5GHPV3 immunogen. CD1 mice (10/group) were primed intramuscularly with either DNA-5GHPV3 (100 µg) or ChAdOx1-5GHPV3 (1 × 10^8^ IU) and then boosted two weeks later with MVA-5GHPV3 (1 × 10^6^ pfu). PBMC responses were determined by IFN-γ Elispot assays at two weeks post-prime and two and three weeks post-boost after stimulation with peptides spanning the entire immunogen sequence, pooled according to protein source. (**A**) Summed response to all pools and (**B**) responses to each peptide pool are shown. (**C**) Frequencies of 5GHPV3-specific CD8+ T cells in splenocytes were analysed three weeks post-boost by ICS in mice vaccinated with CM or DM. Unpaired t test, *p ≤ 0.05. (**D**) Mapping of responses to each segment in 5GHPV3 was performed on splenocytes from 10 CD1 mice vaccinated with CM at two weeks post-boost. Horizontal lines show mean with standard deviation.

As T cell responses in inbred mice are skewed towards a few immunodominant epitopes, we immunised outbred (CD1) mice to enable comparison of the breadth of HPV-specific responses induced by the CM and DM regimens. Priming with DNA-5GHPV3 or ChAdOx1-5GHPV3 alone induced low frequencies of circulating HPV-specific T cells (mean (SD) 3170 (1924) and 13611 (5252) SFU/million PBMCs compared with 3451 and 27574 SFU/million PBMCs in C57BL/6 mice). These responses were strongly boosted by MVA-5GHPV3 to 64734 (64248) and 73933 (68158) SFU/million PBMCs respectively. There was no significant difference in the magnitude of response at either two or three weeks post-boost in the CM versus the DM group (Fig. 5A). However, in contrast to the immunodominance hierarchy observed in C57BL/6 mice, T cell responses in CD1 mice were more evenly distributed across sequences from all six early proteins in the 5GHPV3 immunogen (Fig. 5B, Supplementary Fig. S4).

Intracellular staining of splenocytes at three weeks post-boost indicated a significantly higher frequency of CD8+ IFN-γ+ T-cells in the CM group compared with the DM group (mean (SD) 0.97% (0.37) vs 0.52% (0.51) p = 0.03, Fig. 5C). Polyfunctional HPV-specific CD8+ T cells (CD107, TNF-α and IL2 dual or triple-positive) and CD4+ T cells (IFN-γ, CD107 and IL2 dual or triple-positive) were detected following both regimens. There was no significant difference between the two vaccine regimens in the percentage of polyfunctional T cells.

A critical consideration in our vaccine design was to provide coverage of at least five hrHPV genotypes. To test this, the overlapping peptides corresponding to the 5GHPV3 sequence were re-pooled according to segment and tested in an IFN-γ Elispot assay with splenocytes collected two weeks post-CM immunisation. Mean responses above 25 SFU/million splenocytes (cut-off derived from mean +3 SD of mock-stimulated well values) were observed for 32 of the 59 segments (Fig. 5D). For 57 of the 59 segments at least one mouse per group had a response above this cut-off (Fig. 5D), indicating that almost all segments in the transgene were immunogenic in outbred mice. For example, there are 8 segments in 5GHPV3 that cover regions of E6 from 5 different hrHPV genotypes, of which 7 elicited a positive response two weeks after CM administration.

### T cells specific for sequences in 5GHPV3 are detectable in women with exposure to hrHPV

Next, we wished to confirm that the multi-genotype consensus sequences encoded in 5GHPV3 included epitopes that are recognised by naturally hrHPV-primed T cells. We performed *ex vivo* IFN-γ Elispot assays with PBMC from sexually active women who were selected to represent the spectrum of lifetime exposure to hrHPV: Cohort 1 comprised women aged 16–24 who were predicted to have incident and transient infections; Cohort 2 comprised women aged 25–55 who were predicted to have persistent infection(s). Women were sampled as described in Materials and Methods. As this study is ongoing, we report baseline data only.

**Figure 6:**
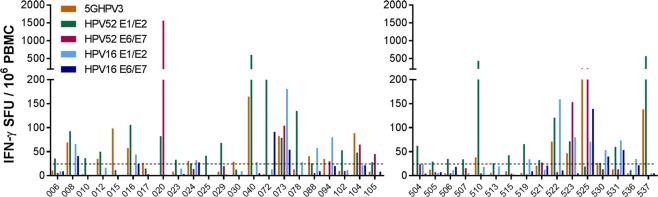
Detection of circulating 5GHPV3-specific T cells by IFN-γ Elispot assays performed in women with prevalent or prior hrHPV infection. PBMCs were collected from healthy asymptomatic women aged 16–24 years; Cohort 1 (left) and women aged 25–55 years referred to colposcopy for investigation of abnormal cervical cytology; Cohort 2 (right). Dotted line at 25 SFU/million PBMCs shows negative cut-off (calculated as mean of negative wells plus 3 SD). Study participants shown in the graphs are those that had a detectable response (>25 SFU/million PBMCs) to either of the reference peptide sets.

HrHPV prevalence at study entry was 25% in Cohort 1 (27/106 women with available data; all non-vaccine types) and 88% of women had received prior prophylactic vaccination (Supplementary Table S3). Twenty-two of 101 women analysed by IFN-γ Elispot showed responses >25 SFU/million PBMC to one or more pools of hrHPV peptides based on early protein sequences (E1, E2, E6, E7) from two reference genotypes: HPV16, and HPV52, the most prevalent hrHPV type in vaccinated women in the UK^33^ (Fig. 6). These responses indicate past or current infection with hrHPV infection(s), since the early proteins are not a component of prophylactic vaccines and 14/22 women were positive for hrHPV DNA at the time of blood sampling. Nine of the 22 women with E1/E2/E6/E7 responses (41%) also recognised the 5GHPV3 peptides. In addition, three women made responses to 5GHPV3 but not to any of the HPV16 or HPV52 early proteins, consistent with exposure to genetically distant hrHPV types.

In Cohort 2, baseline hrHPV prevalence was 89% (31/35 women with available data; predominantly non-vaccine types) and 8% had received prior prophylactic vaccination (Supplementary Table S3). Twenty-five (71%) had biopsy-confirmed cervical abnormalities (14 HPV change only, 10 CIN 1/2, 1 CIN 3). Fifteen women (43%) showed responses >25 SFU/million PBMC to one or more pools of E1, E2, E4, E5, E6 and E7 peptides. Six of these 15 (40%) women had detectable T cell responses to 5GHPV3 (Fig. 6). Using reactivity to reference hrHPV peptide pools as the denominator, the difference in proportions of responders to 5GHPV3 peptides between the two cohorts was not statistically significant.

## Discussion

The high degree of genetic distance among hrHPV types and their intrinsic capacity to avoid immune detection pose significant challenges for the development of immunotherapy for pre-cancerous cervical lesions. Here we developed a multi-genotype, multi-protein immunogen, 5GHPV3, which is composed of sequence fragments that are conserved and weighted to ensure geographic representation of the global hrHPV population at the protein level. We used a potent heterologous viral vector platform to demonstrate the immunogenicity of 5GHPV3 in inbred and outbred mice and confirmed that T cell responses were elicited to each genotype and to each of the 6 early proteins. The detection of polyfunctional HPV-specific CD8+ and CD4+ effector T cells in the cervix as well as the circulation confirmed that systemic administration of the ChAdOx1 and MVA vectors induces T cells with tissue-homing capacity. Furthermore, these increased in frequency over time, indicating continued trafficking to the cervix, and upregulated α4β1 integrin. In parallel, we detected 5GHPV3-reactive T cells *ex vivo* in women with current or past hrHPV exposure, confirming that 5GHPV3 contains sequences that are immunogenic during the course of natural hrHPV infections.

The most advanced therapeutic vaccine strategies for hrHPV are based on immunogens comprising full length or near-full length viral proteins, derived from a limited number of viral sequences^22,25^. Bioinformatic approaches using conservation analysis have been described previously, albeit with a low conservation threshold and without sequence normalisation to account for variation in global genotype prevalence, nor experimental validation of the approach^34^. Ragonnaud *et al*. used phylogenetics to construct ancestral papillomavirus sequences for E1 and E2 and demonstrated their immunogenicity *in vivo* using a replication-deficient adenovirus for delivery^24^. Our conservation algorithm generated multiple conserved regions across five hr genotypes that cause both CIN and cancer^35^. However, as they show distinct phylogenetic clustering it was necessary to use the complementary approaches of creating chimeric sequences and selecting variants to ensure coverage. A limitation of our vaccine is that not all hrHPV types could be represented. For example, hrHPV 45 is highly prevalent in sub-saharan Africa. As it is closely related to HPV18, this could be addressed by the inclusion of an HPV 45/18 consensus sequence in further iterations of the immunogen.

We hypothesised that induction of immune responses to all early proteins will be necessary to improve on the clinical efficacy of therapeutic vaccines that target E6, E7 and/or E2. E6 and E7 are constitutively expressed in both premalignant and invasive lesions and the concerted actions of these proteins result in malignant transformation of HPV-infected cells and uncontrolled tumour growth. They are therefore essential targets for immunotherapeutics. However, while various studies have reported circulating T cell responses to E6 and E7 in hrHPV-exposed women, they have been associated with both clearance and persistence of cervical lesions, whereas E2-specific T cell responses have a stronger association with CIN regression and viral clearance^19,20^. Furthermore, an MVA-vectored vaccine encoding E2 was reported to cure high grade CIN and HPV infection in a large uncontrolled trial^36^. Since both E1 and E2 are expressed at higher levels than E6 and E7 early in the progress of an HPV infection, these proteins may also serve as good targets for therapeutic vaccines^37,38^. Paolini *et al*. reported the detection of E5 transcripts in nearly 70 clinical samples, including lesions with HPV-related changes and low or high-grade abnormalities, indicating a possible biological activity of E5 products^26^. Given the contribution of E5 to malignant progression it may also be an important target. We have demonstrated that the 5GHPV3 immunogen elicited responses to all early proteins in outbred mice, therefore, it will be possible to evaluate the therapeutic benefit of targeting the less well-studied early proteins in clinical trials.

The use of replication-defective viral vectors, ChAdOx1 and MVA for antigen delivery has advantages over recombinant DNA and sub-unit vaccines. The latter are poorly immunogenic in humans unless adjuvanted and do not prime CD8+ T cells efficiently, whereas replication-defective adenoviruses prime strong CD8+ and CD4+ T cell responses^39–41^. We confirmed that vaccine-induced HPV-specific CD8+ and CD4+ T cells have lytic capacity and a T helper phenotype respectively, which are considered to be necessary for mediating regression of HPV lesions and viral clearance. Our results are consistent with those from Kaufman *et al*. who showed that intramuscular immunisations with adenovirus-vectored SIV Gag in mice and monkeys elicited CD8+ T cells that migrated rapidly from systemic to mucosal compartments to generate potent and durable mucosal immune responses^42,43^.

To date, we have analysed T cell responses to 5GHPV3 peptides in hrHPV-exposed women using *ex vivo* assays only. A higher response rate may be detected using cultured assays, given that HPV-specific T cell responses are often transient and of low magnitude^19,44^. An additional limitation is that we focused on responses in the circulation, for logistical reasons. Cervical sampling to capture tissue-resident T cells should be considered for future studies.

The TC-1 tumour challenge model has been used extensively to evaluate the anti-tumour efficacy of therapeutic HPV vaccines in C57BL/6 mice. TC-1 tumours are easily prevented or controlled by T cell responses to one immunodominant H-2D^b^–restricted E7 epitope, RAHYNIVTF, which may constitute more than 22% of the entire CD8+ T cell compartment^45–48^. We did not use this model here because this epitope was not included in 5GHPV3. Furthermore, the TC-1 model does not allow interrogation of the therapeutic potential afforded by T cell responses to early proteins other than E6 and E7 nor from genotypes other than HPV-16.

In summary, we systematically designed an immunogen to elicit T cell responses to all the early HPV proteins, with the aim of interrupting progression of persistent cervical infections and disease. This strategy could also be considered for treatment of preinvasive anal HPV disease, which is under-diagnosed and is frequently present in women with cervical lesions^49^. The heterologous viral vector platform has key advantages over conventional approaches, namely, the capacity to elicit potent mucosal immune responses after systemic administration and excellent human safety and tolerability. Furthermore, ChAdOx1 is serologically distinct from common circulating human adenoviruses and therefore unlikely to be impacted by pre-existing Ad-specific antibodies. Manufacture of these vaccine candidates under GMP conditions is now underway, in preparation for clinical trials, which will evaluate safety, tolerability and impact on rates of viral clearance and regression of cervical and anal intraepithelial lesions.

## Supplementary information

Supplementary information

## Data Availability

The datasets generated and analysed during the current study are available from the corresponding author on reasonable request.
