# Supplementary material for: A multi-genotype therapeutic human papillomavirus vaccine elicits potent T cell responses to conserved regions of early proteins
Source: Sci Rep. 2019 Dec 10;9:18713. doi: 10.1038/s41598-019-55014-z (PMC6904585; doi:10.1038/s41598-019-55014-z)
Supplement: Supplementary file 1 — Supplementary information [file 41598_2019_55014_MOESM1_ESM.pdf]

A multigenotype therapeutic human papillomavirus vaccine elicits potent T cell responses to conserved regions of early proteins

Gemma Hancock, Joshua Blight, Cesar Lopez-Camacho, Jakub Kopycinski, Mamatha Pocock, Wendy Byrne, Michael J Price, Phillip Kemlo, Ranoramanana Evans, Kathryn Saunders, Richard Kirton, Monique Andersson, Karin Hellner, Arturo Reyes-Sandoval, Lucy Dorrell

Supplementary Figure 1

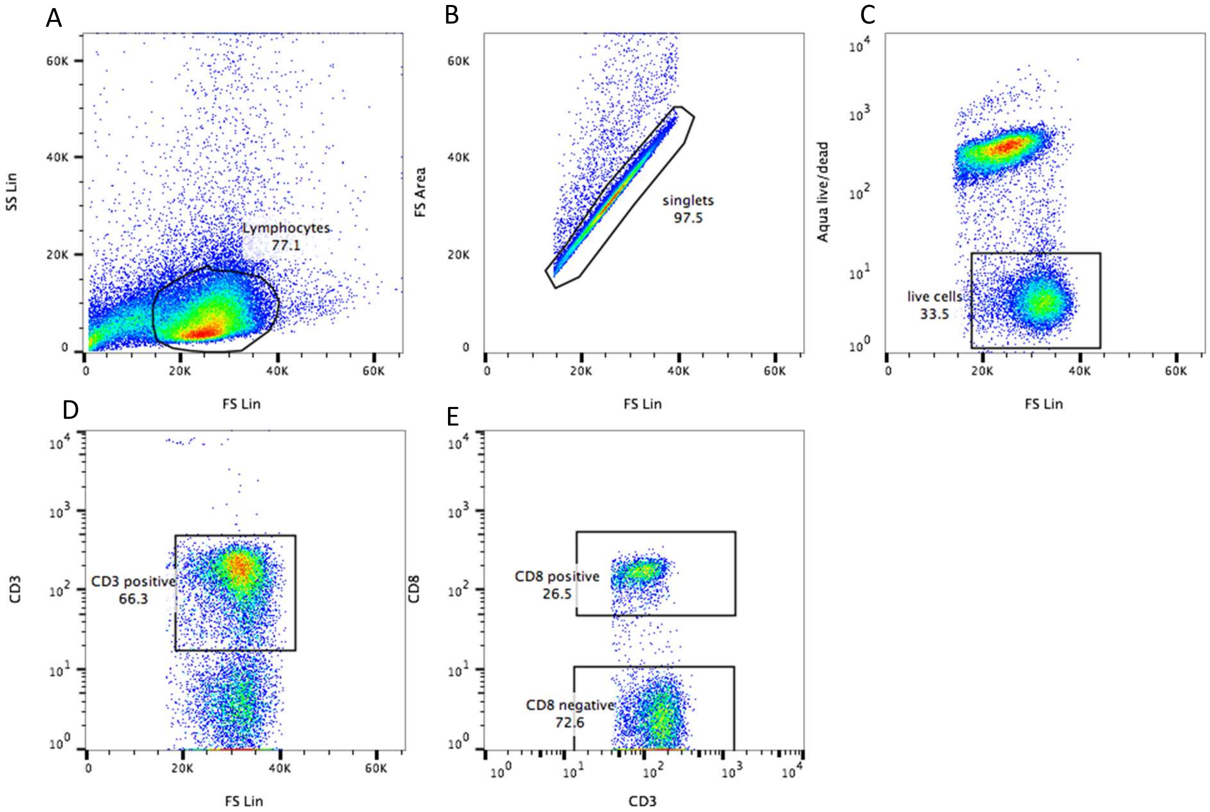

Gating strategy for ICS. Single cell suspensions of PBMCs, splenocytes or cervicovaginal lymphocytes were prepared and stimulated for 6.5 hours with 5GHPV3 peptide pools E1, E2, E4/5, E6 and E7 (10µg/ml), negative control (0.1% DMSO) and positive controls (SEB, 10µg/ml). Following surface and intracellular staining cells were acquired using a CyAn flow cytometer. Lymphocytes (A) were gated on FS lin and SS lin, singlets (B) were gated on FS lin and FS area, live cells (C) were gated on FS lin and aqua live/dead, CD3+ cells (D) were gated on FS lin and CD3 and CD8 and CD4 cells (E) were gated on CD3 and CD8.

Supplementary Figure 2

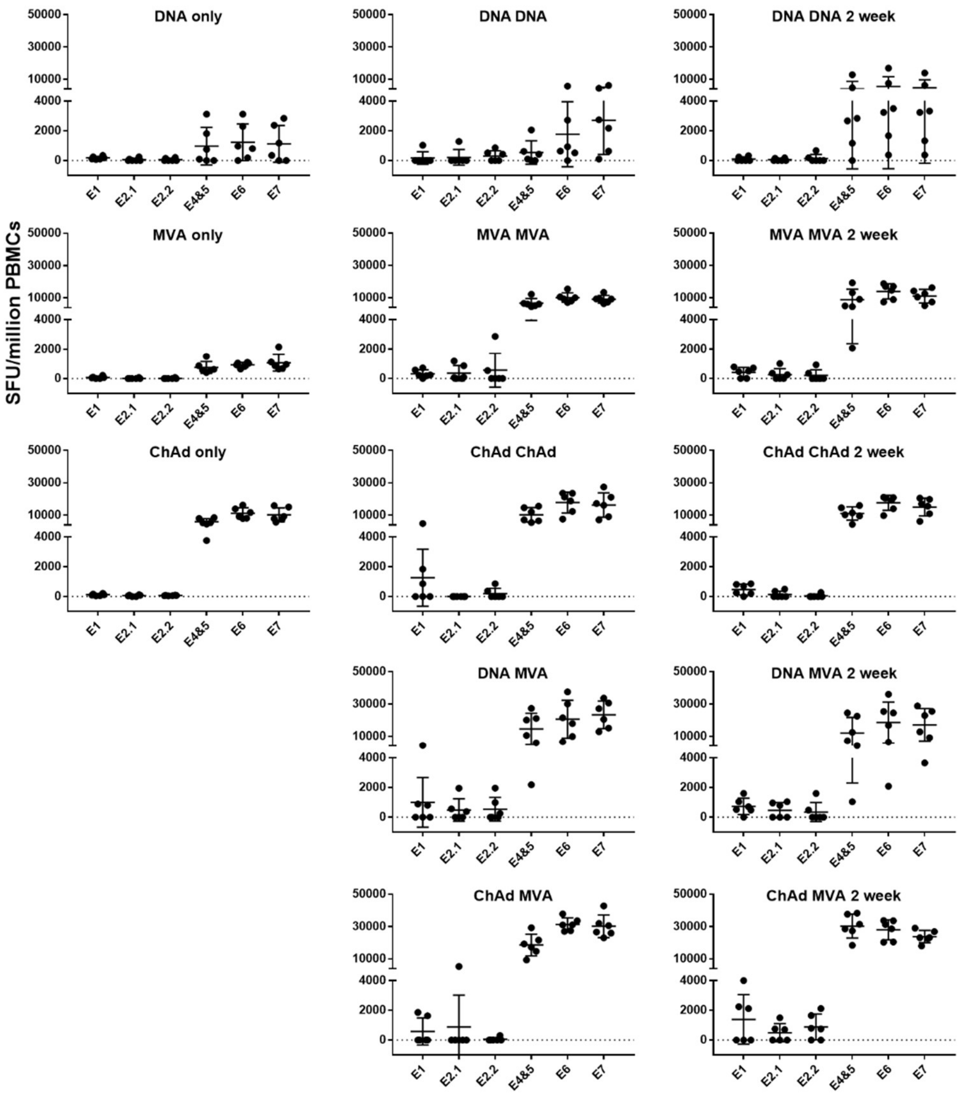

Induction of T cell responses to 5GHPV3 administered in different vaccination regimens, as measured by IFN-γ Elispot. C57BL/6 mice (6/group) were primed intramuscularly with DNA-5GHPV3 (100µg), MVA-5GHPV3 (1x106 pfu) or ChAdOx1-5GHPV3 (1x108 IU) and then boosted intramuscularly with a heterologous or homologous vaccine two weeks later. Tail vein bleeds were performed two weeks post-prime and one and two weeks post-boost. PBMCs were stimulated with peptides spanning the entire immunogen sequence, pooled according to protein source. Data show responses to each peptide pool. Horizontal lines show mean with standard deviation.

Supplementary Figure 3

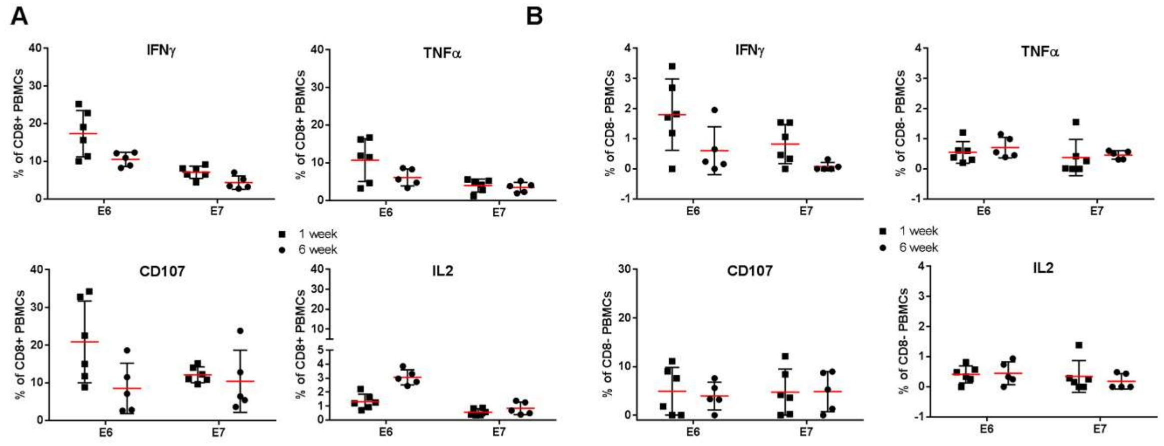

ICS was performed on PBMC from C57BL/6 mice (6/group) vaccinated with the CM regimen at one and six weeks post-boost following stimulation with 5GHPV3 E6 and E7 peptide pools. Data shown are CD8+ T cells (A) and CD8-T cells (B).

Supplementary Figure 4

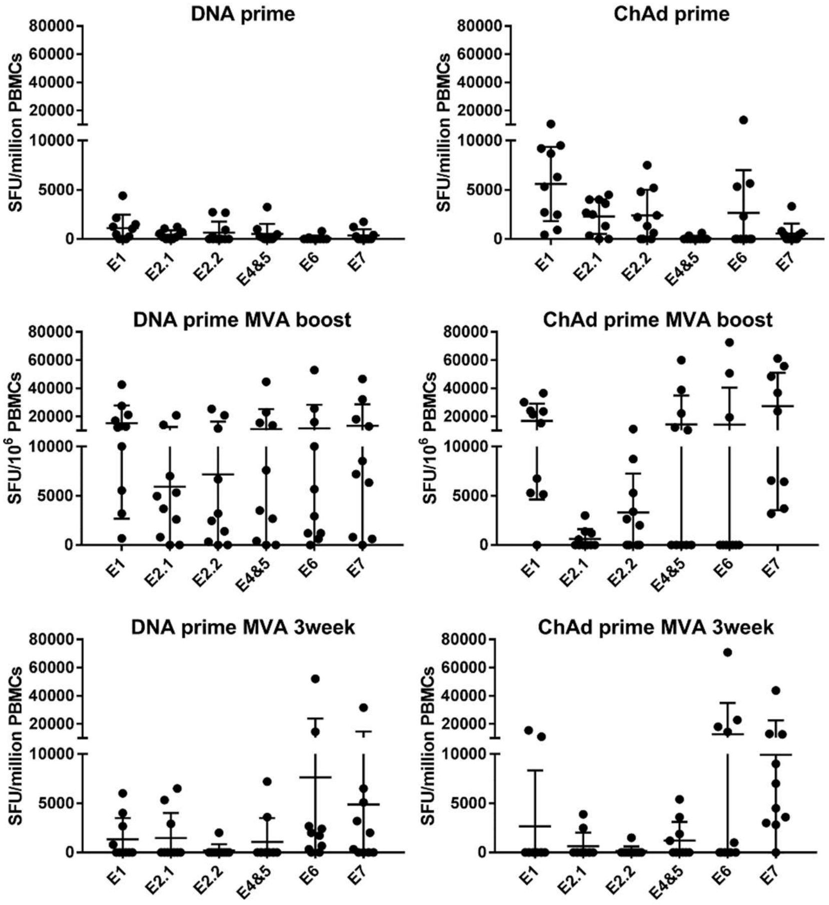

CD1 mice (10/group) were primed intramuscularly with either DNA-5GHPV3 (100µg) or ChAdOx1-5GHPV3 ($1x10^8$ IU) and then boosted two weeks later with MVA-5GHPV3 (1x106 pfu). PBMC responses were determined by IFN-γ Elispot assays at two weeks post-prime (top) and two (middle) and three (bottom) weeks post-boost after stimulation with peptides spanning the entire immunogen sequence, pooled according to protein source. Responses to each peptide pool are shown.

Supplementary Table 1

Number of full length protein sequences collected from NCBI protein database of high quality sequencing

Protein	Genotype				
	**16**	**18**	**31**	**52**	**58**
E1	126	49	24	27	53
E2	195	56	26	32	54
E4	197	48	24	24	60
E5	1205	78	90	218	185
E6	566	70	85	193	199
E7	126	49	24	27	53

.

Supplementary Table 2

Reagent panel for intracellular cytokine staining

**Specificity**	**Clone**	**Fluorochrome**	**Supplier**
CD3	17A2	Alexa Fluor 750	eBioscience
CD8	53-6.7	PerCP Cy5.5	BioLegend
CD4	RM4-5	PECF594	BD Bioscience
CD29	HM β1-1	PE (phycoerythrin)	BD Bioscience
CD49	R1-2	FITC	BioLegend
CD44	IM7	BB515	BD Bioscience
CD62L	MEL-14	PE (phycoerythrin)	BD Bioscience
CD127	SB/199	BV421	BD Bioscience
IFN-γ	XMG1.2	APC	BD Bioscience
IL-2	JES6-5H4	PE	BD Bioscience
TNFα	MP6-XT22	FITC	BioLegend
CD107	1D4B	BV421	BD Bioscience

Supplementary Table 3

Baseline characteristics of study participants

	**Cohort 1**	**Cohort 2**
**n**	106	34
**Age, mean (range)**	21 (16-24)	32 (25-47)
**Vaccinated (at least 1 dose, bi/quadrivalent)**	93 (88%)	7 (21%)
**Baseline HR HPV DNA positive**	27[a] (25%)	31[b] (91%)
**Age at sexual debut, mean (range)**	17 (14-24)	17 (14-29)

a All hrHPV were Other (non-16, non-18) types

b hrHPV types were: Other (n = 23), Other + HPV16 (n = 3), Other + HPV18 (n = 1), HPV16 only (n = 2), HPV18 only (n = 2)
